# Optimization of Photopolymer Materials for the Fabrication of a Holographic Waveguide

**DOI:** 10.3390/polym9090395

**Published:** 2017-08-26

**Authors:** Cristian Neipp, Jorge Francés, Francisco J. Martínez, Roberto Fernández, Mariela L. Alvarez, Sergio Bleda, Manuel Ortuño, Sergi Gallego

**Affiliations:** 1 I.U. Física Aplicada a las Ciencias y las Tecnologías, Universidad de Alicante, P.O. Box 99, E-03080 Alicante, Spain; jfmonllor@ua.es (J.F.); fj.martinez@ua.es (F.J.M.); roberto.fernandez@ua.es (R.F.); mariela.alvarez@ua.es (M.L.A.); sergio.bleda@ua.es (S.B.); mos@ua.es (M.O.); sergi.gallego@ua.es (S.G.); 2 Department de Física, Ing. de Sistemas y Teoría de la Señal, Universidad de Alicante, P.O. Box 99, E-03080 Alicante, Spain

**Keywords:** photopolymers, diffraction gratings, holographic waveguide, holography

## Abstract

In this work, we present a method of manufacturing an optical see-through display based on a holographic waveguide with transmission holograms that couple the incident light between air and the glass substrate, accomplishing total internal reflection. The holograms (slanted transmission gratings with a spatial frequency of 1700 lines/mm) were recorded on a polyvinyl alcohol acrylamide (PVA/AA) photopolymer. We will also show that the addition of *N*,*N*′-methylene-bis-acrylamide (BMA) to the composition of the photopolymer allows the achievement of the index modulations necessary to obtain high diffraction efficiencies in non-slanted diffraction gratings of 1000 and 2200 lines/mm, and also in slanted gratings of 1700 lines/mm (which are the base of the optical system proposed).

## 1. Introduction

Recently, augmented reality or virtual reality has burst with force in society, allowing the creation of video games scheduled to play with virtual reality glasses or the manufacture of glasses that act as mobile devices connected to the Internet (“Google glass”). Augmented reality devices usually consist of a headset and a display system to show the user the virtual information that is added to reality. The two main display systems used are the optical see-through display and the video-mixed display. Both use virtual images that are displayed to the user, mixed with reality or projected directly on the screen. On the other hand, the “glasses” have some advantages in relation to a mobile direct-viewing screen, for instance hands-free and high privacy characteristics. Among the different models of glasses proposed [1,2], see-through glasses are especially important for mobile use because they are safer and more easily accepted by the public. However, combining high-quality images with clear visibility through the whole system remains a challenge.

In recent years, planar waveguide technology with holographic optical elements has been widely used in such devices [3,4,5]. This technology could drastically reduce the size and weight of display systems [6,7,8]. However, there are still some technical difficulties when manufacturing such devices. One of them is due to the chromatic dispersion of the holographic optical elements (HOE) [9], so it is complex to clearly reproduce a chromatically rich image in the exit plane. In addition, the planar waveguides are generally fabricated with reflection holographic optical elements. If one intends to use photopolymers as recording materials, to take advantage of their main features (such as dynamical chemical composition, the possibility of introducing many new components, and low cost), it is necessary to take into account the possible limitations at high spatial frequencies. This is because at high spatial frequencies and thus small periods, the non-local effects begin to have greater importance. There are different ways to tackle this problem. One possibility is to optimize the compositions of the polymers to work at high spatial frequencies. Different authors have worked in this direction; for example, Fuentes et al. introduced chain shorteners such as 4,4′-Azobis (4-cyanopentanoic acid) (ACPA) [10], to improve the response of photopolymers for the recording of diffraction gratings by reflection. D. Cody et al. [11] reported the addition in the composition of a chain transfer agent and a free radical scavenger to enhance the holographic recording ability of a diacetone acrylamide (DA)-based photopolymer for reflection, obtaining values of diffraction efficiency higher than 50%. For holographic transmission gratings, but with high spatial frequencies (3000 lines/mm), Zhu et al. [12] demonstrated that, by employing a low molecular weight polyvinyl alcohol (PVA) as a binder and increasing the ambient recording temperature, diffraction efficiencies of 94% can be achieved for red-sensitive PVA/acrylamide-based photopolymer. Another option is to design the planar waveguide in a different way using transmission holograms, in which case it is easier to optimize the composition of the recording material. For slanted transmission gratings, different authors reported high values of diffraction efficiency: Vojtíšek et al. demonstrated overmodulation effects in highly efficient slanted transmission gratings produced in photopolymer recording material Bayfol HX [13]; Yu et al. studied the influence of the relative humidity on the diffraction efficiency of slanted transmission gratings with 3000 lines/mm, also obtaining high values of diffraction efficiency for the optimum conditions [14]. In this paper, we present a method of manufacturing an optical see-through display based on a holographic waveguide with transmission holograms. The system to be developed consists mainly of three parts: a holographic optical element that couples the input light to the waveguide, another holographic optical element that couples the light from the waveguide to the outside, and the waveguide itself. The glass that acts as a substrate for the holographic material will act as a waveguide, so that the optimization of the system focuses on the two holographic elements that act as couplers.

To produce the transmission holograms of the optical see-through display, a polyvinyl alcohol acrylic photopolymer (PVA/AA) was used [15]. In order to understand the capabilities of this material for this particular application, non-slanted transmission holographic gratings were recorded with spatial frequencies of 1000 and 2200 lines/mm. Subsequently, slanted gratings with a spatial frequency of 1700 lines/mm, which are the basis of the device, were recorded and analyzed.

This work is divided as follows: firstly the device proposed to act as a holographic waveguide is explained. Secondly, the composition of the photopolymer and the experimental setup is presented. Finally, the results obtained are discussed.

## 2. Theoretical Design

**Figure 1:**
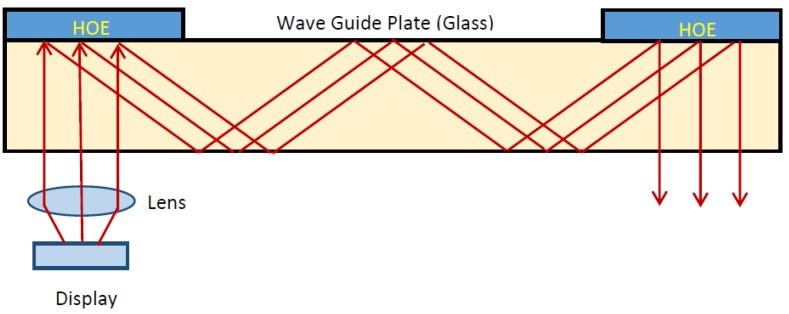
Holographic waveguide by two reflection holograms.

In this section, we will explain the design parameters of the holographic waveguide. The configuration generally used for devices of this type is shown in Figure 1. As can be seen, two reflection holograms are used to achieve the total reflection condition. The first hologram in front of the lens couples the light to the waveguide in total reflection condition, while the second hologram couples the beam out of the waveguide. As discussed in the introduction, although this is the system commonly used for these applications, there are limitations in the manufacture of reflection holograms in photopolymers as a consequence of the high spatial frequencies, since one must carefully adjust the compositions of the photopolymers.

**Figure 2:**
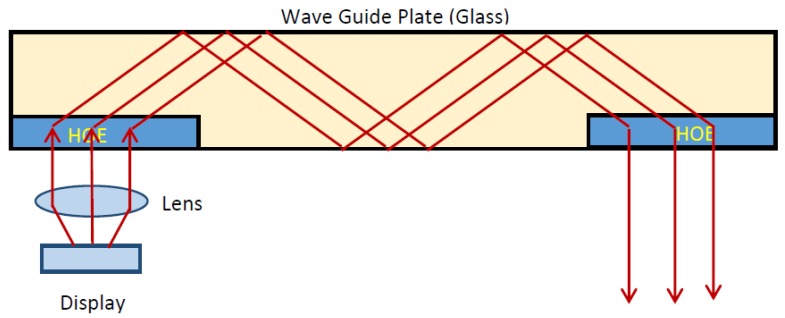
Holographic waveguide by two transmission holograms.

The device we propose based on two transmission holograms is shown below (Figure 2).

The main feature is that, in changing from reflection to transmission holograms, the holograms are recorded with lower spatial frequencies, which permits flexible working conditions; in particular, the compositions of the photopolymers can be chosen with less stringent conditions.

**Figure 3:**
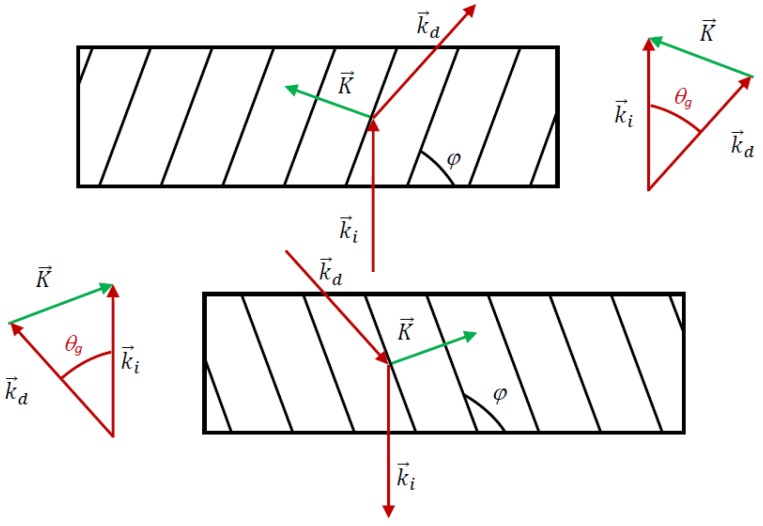
Couple-in and couple-out diffraction gratings.

In order to work as couplers, the transmission holographic gratings should couple the incident light between air and the glass substrate, accomplishing total internal reflection. To perform this task, the configuration of the gratings is illustrated in Figure 3, where it is important to notice that the diffracted beam should form a critical angle (total internal reflection) with respect to the normal. ${\vec{k}}_{i}\;$and ${\vec{k}}_{d}$ are the propagation vectors of the incident and diffracted beam, respectively, $\vec{K}$ is the grating vector and θ_g_ is the critical angle inside the photopolymer.

For the particular case of red light, wavelength λ_r_ = 633 nm, and assuming an average refractive index of the photopolymer of *n*_g_ = 1.478, the critical angle in the photopolymer is θ_g_ = 42.57°. For the glass substrate, assuming a refractive index of 1.51, the critical angle is θ_c_ = 41.47°.

The grating vector $\vec{K}$ can be calculated as:

(1) $$\vec{K}={\vec{k}}_{i}-{\vec{k}}_{d}$$

The grating vector is related to the spatial frequency, *f*, and the period of the grating, Λ, by:

(2) $$\left|\vec{K}\right|=2\pi f=\frac{2\pi }{\Lambda }$$

Taking into account that

(3) $$\left|{\vec{k}}_{i}\right|=\left|{\vec{k}}_{d}\right|=\frac{2\pi }{\lambda_{r}}n_{g}$$

and that the directions of the propagation vectors are those shown in Figure 3, the diffraction grating should have a spatial frequency of 1690 lines/mm. The angle ϕ formed by the interference fringes with the substrate can be calculated by using:

(4) $$\varphi =\mathrm{atan}\left(\frac{K_{y}}{K_{x}}\right)-\frac{\pi }{2}$$

In this case ϕ = 69.3°.

**Figure 4:**
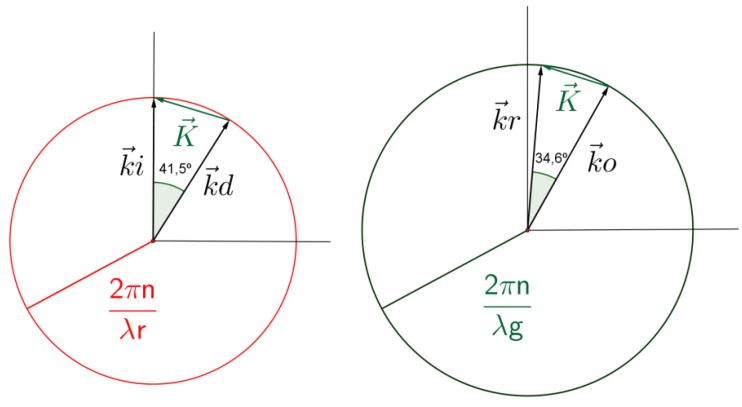
Recording and reconstruction geometry.

Although we have found the design parameters of the gratings to accomplish total internal reflection, it is clear that these grating cannot be recorded using red light since the angle of the object beam should be π/2 in air. Therefore, the grating should be fabricated with a shorter wavelength. In this work, the gratings were recorded with green light, λ_g_ = 532 nm. In Figure 4, the conditions of reconstruction (left figure) and recording (right figure) are shown. To record a grating with the required grating vector with a wavelength of 532 nm, the angles of the reference and object propagation vectors with respect to the normal should be: in air, θ_r_ = 5.2° and θ_o_ = 68.5°; and in the photopolymer, θ_r_ = 3.42° and θ_o_ = 38°.

## 3. Experimental Setup

In order to record the holographic transmission gratings that constitute the coupled-in and coupled-out holograms in the photopolymer material, the material must possess the following characteristics: it must be sensitized to green color; index modulations yielding to high values of diffraction efficiency must be able to be recorded on it; the fringes must have sufficient stability in the slanted diffraction gratings; and it must undergo small thickness variations after the recording process. In this work, we use PVA/AA photopolymer, which is composed of acrylamide (AA) as a polymerizable monomer, *N*,*N*′-methylene-bis-acrylamide (BMA) as a crosslinking monomer, triethanolamine (TEA) as a co-initiator and plasticizer, yellowish eosin (YE) as a dye, polyvinyl alcohol (PVA) as a binder, and a small proportion of water as an additional plasticizer. Different types of PVA can be used as a binder; in particular, the quantity of water retained inside the layer depends on the molecular weight of the PVA. That is, the higher the molecular weight, the more water will remain in the final layer; thus, this can affect the stability of the slanted gratings required for the application proposed in this paper [16]. In this work, we used a PVA 18-88 with *M*_w_ = 180,000 u looking for high values of remaining water, a more stable layer at ambient conditions, and faster values of monomer diffusion [17]. In particular, let us introduce the role of the crosslinker for this application; on the one hand, the role of the crosslinker is to increase the polymer chain length to give more structural strength to the phase hologram. Nevertheless, for long polymer chains, the effect of non-local polymerization [18] increases the difficulty of the recording of high resolution holograms [12], such as that required in the present application. In this sense, it is important to find a balance, thus, we evaluated the behavior of the material at 2200 lines/mm. The particular concentration used in this work is presented in Table 1.

For the preparation of the layer, 30 mL of a solution with water as the solvent was deposited, using the force of gravity, on a glass substrate (25 cm × 20 cm) and left in the dark (RH = 40–45%, *T* = 20–23 °C). When part of the water had evaporated (after about 36 h), the layer had enough mechanical resistance and could be cut without deforming. The final “solid” film had a physical thickness around 85 ± 10 µm. This final thickness can be modified by changing the quantity of the syrup deposited on the glass. The refractive index was measured before exposure using a refractometer, obtaining a value of 1.4811.

**Figure 5:**
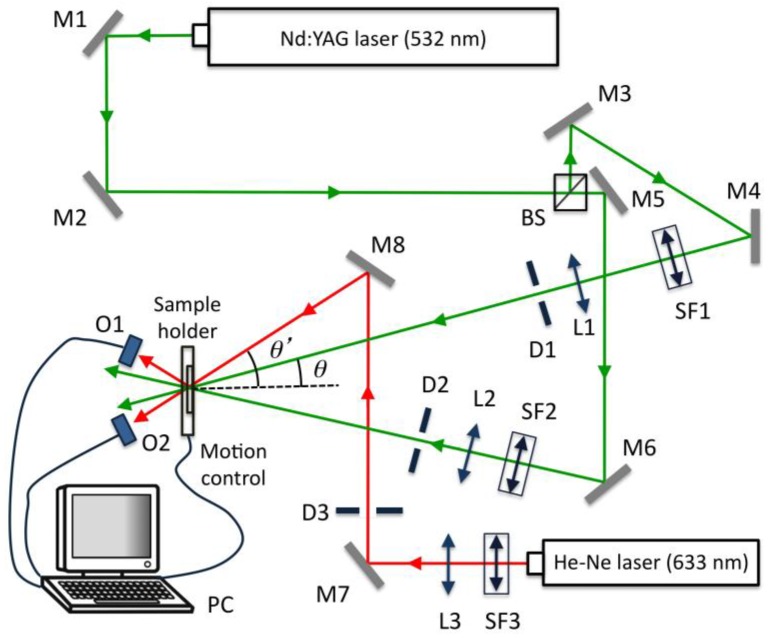
Experimental setup. BS: Beamsplitter; M*i*: mirror, SF*i*: spatial filter; L*i*: lens; D*i*: diaphragm; O*i*: optical power meter; PC: data recorder.

The experimental device is a typical transmission holographic setup; it is represented in Figure 5. A Nd:YAG laser tuned at a wavelength of 532 nm was used to record diffraction gratings by means of continuous laser exposure. The laser beam was split into two secondary beams with an intensity ratio of 1:1. The diameter of these beams was increased to 1 cm using a spatial filter and collimating lens, while spatial filtering was ensured. The working intensity at 532 nm was 3 mW/cm^2^. Non-slanted diffraction gratings were recorded with two different spatial frequencies. In the first case, the object and reference beams were recombined at the sample at an angle of 15.2 degrees to the normal with an appropriate set of mirrors, and the spatial frequency obtained was 1004 lines/mm. In the second case, the beams were recombined at the sample at an angle of 36.6 degrees to the normal, obtaining gratings of 2200 lines/mm. Slanted gratings of 1700 lines/mm were also recorded; to do this, the reference beam formed an angle to the normal of −5.2 degrees, whereas the object beam formed an angle of 68 degrees.

We monitored the diffraction grating using red light (λ = 633 nm), which the dyes do not absorb. After recording, the sample was rotated to record the angular response around the first Bragg condition.

## 4. Results and Discussion

**Figure 6:**
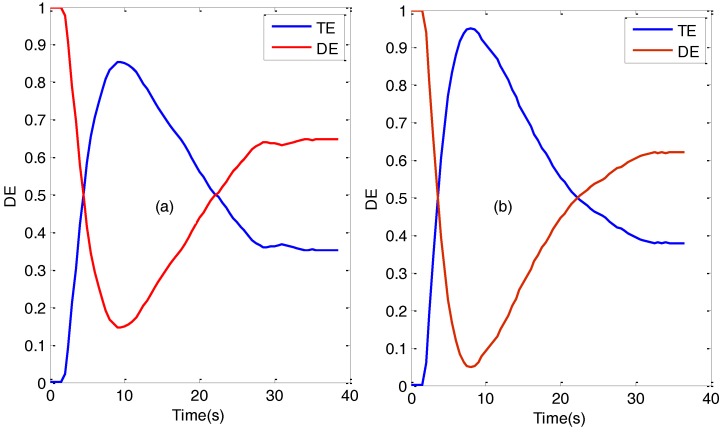
Diffraction and transmission efficiency as a function of the time of exposure for non-slanted gratings. (**a**) For a 2200 lines/mm transmission grating; (**b**) For a 1000 lines/mm transmission grating.

Firstly, we analyzed the diffraction efficiency, DE, and transmission efficiency, TE, versus recording time for non-slanted gratings recorded in PVA/AA with two spatial frequencies: 1000 and 2200 lines/mm. The results are shown in Figure 6. It can be seen that diffraction efficiencies as high as 90% were obtained in both cases. As commented in the last section, in this work we added BMA as a crosslinking monomer in order to guarantee the stability of the fringes, particularly in the case of the slanted gratings. It is well known that the crosslinker BMA causes a high index modulation [19,20,21], so in this case the highest diffraction efficiency was achieved at a relatively short time exposure. It is also important to note that in both cases, after reaching the maximum diffraction efficiency, the diffraction efficiency decayed rapidly. This decay, as will be seen later, is due to overmodulation. This fact demonstrates the ability of BMA to increase the modulation of the refractive index. However, after the peak diffraction efficiency there is no flat response, so special care must be taken in choosing the optimum exposure to record the hologram. Another interesting fact is that, for the particular gratings studied, the absorption of the material is sufficiently low.

**Figure 7:**
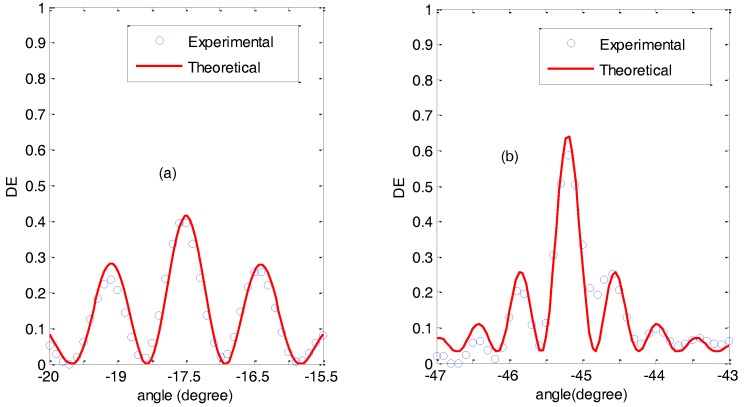
Diffraction efficiency as a function of the angle for non-slanted recorded gratings. (**a**) Diffraction grating of 1000 lines/mm; (**b**) Diffraction grating of 2200 lines/mm.

The angular response of the diffraction efficiency was also monitored as a function of the angle for non-slanted gratings recorded in PVA/AA photopolymer. In Figure 7 the experimental data and the theoretical fits using Kogelnik’s coupled wave theory [22] are represented. In both cases (both spatial frequencies), overmodulation effects can be observed [23].

To gain a better idea of these overmodulation effects, the product Δ*nd* (Δ*n* being the refractive index modulation and *d* being the thickness of the grating) yielding to the maximum theoretical diffraction efficiency was calculated using Kogelnik’s Theory. For non-slanted diffraction gratings, the diffraction efficiency, η, at Bragg angle can be obtained as [22]:

(5) $$\eta =sin^{2}\left(\frac{\pi \Delta nd}{\lambda cos\theta \prime_{B}}\right)$$

where λ is the reconstruction wavelength in vacuum and θ′_B_ is the Bragg angle in the photopolymer. Therefore, the product Δ*nd* yielding to the maximum diffraction efficiency can be calculated as:

(6) $$\Delta nd=\frac{\lambda cos{\theta \prime }_{B}}{2}$$

For a spatial frequency of 1000 lines/mm this value is Δ*nd* = 0.309 μm, whereas for 2200 lines/mm Δ*nd* = 0.277 μm. The fittings of Figure 7 gave a result of Δ*nd* = 0.430 μm in the case of the grating with 1000 lines/mm and Δ*nd* = 0.396 μm for the grating of 2200 lines/mm. It is thus clear that the gratings recorded in PVA/AA with BMA as a crosslinker are overmodulated. Nonetheless, a diffraction efficiency higher than 60% was obtained in both cases.

Finally, we analyzed slanted gratings recorded with a spatial frequency of 1700 lines/mm. In this case, the diffraction efficiency at Bragg angle is calculated as:

(7) $$\eta =sin^{2}\left(\frac{\pi \Delta nd}{\lambda \sqrt{c_{r}c_{s}}}\right)$$

where *c_r_* and *c_s_* are the cosine of the angles that the reference and object beam, respectively, form with the normal of the grating. The product Δ*nd* yielding to the maximum diffraction efficiency can be calculated as:

(8) $$\Delta nd=\frac{\lambda \sqrt{c_{r}c_{s}}}{2}$$

In the particular case of the gratings recorded in this work, Δ*nd* = 0.281 μm.

**Figure 8:**
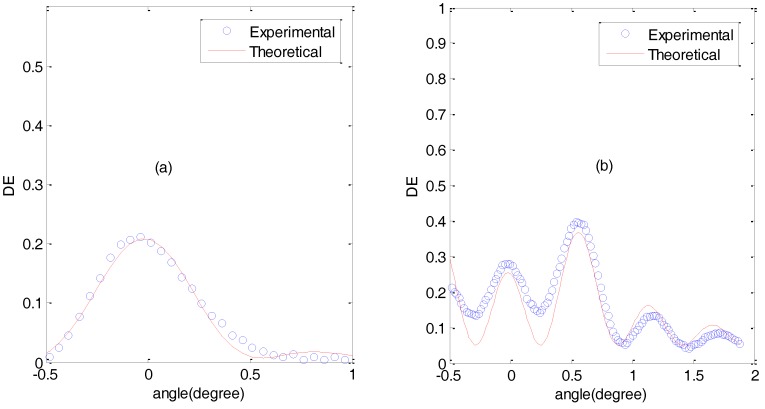
Diffraction efficiency as a function of the angle for a slanted recorded grating of 1700 lines/mm. (**a**) Undermodulated grating; (**b**) Overmodulated grating.

Figure 8 shows the angular response of the diffraction efficiency for a slanted grating of 1700 lines/mm in two cases: Figure 8a represents an undermodulated grating, whereas Figure 8b represents an overmodulated grating. For the undermodulated grating, the fitting gave a result of Δ*nd* = 0.081 μm, whereas in the case of the overmodulated grating, the fitting gave a result of Δ*nd* = 0.45 μm. In the case of the overmodulated grating, there are two aspects that should be addressed: firstly, the fact that the side lobes are higher than the main lobe (centered at the Bragg angle, 0 degree in this case) is a typical behaviour of overmodulated gratings [23]; secondly, the deviation of the experimental data with respect to the theoretical curve, in particular the fact that the theoretical curve remains below the experimental data from 0 to 1 degree, is due to a refractive index modulation attenuated in depth [24]. In any case, it is important to highlight that with the proposed composition, the required refractive index modulation for high diffraction efficiencies can be obtained in slanted gratings with a spatial frequency of 1700 lines/mm.

**Figure 9:**
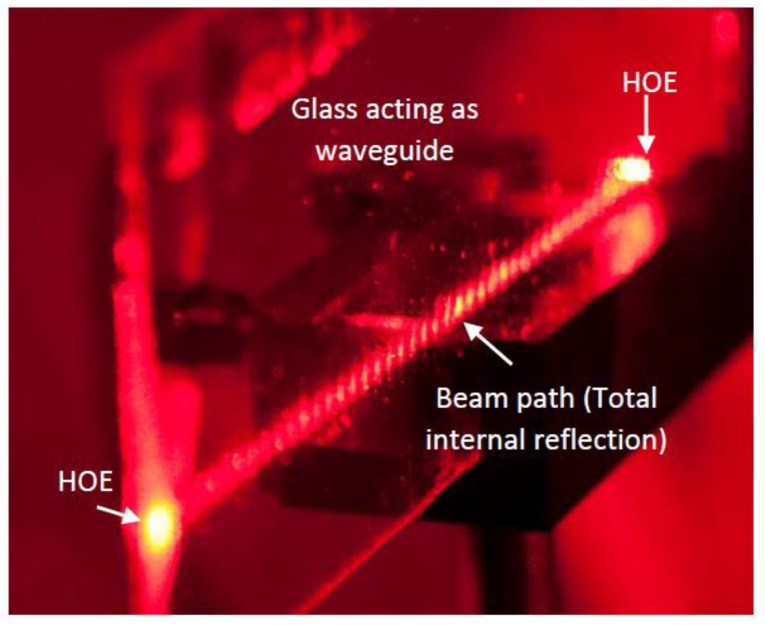
Holographic waveguide.

We finally present in Figure 9 the holographic waveguide fabricated with the non-slanted gratings of 1700 lines/mm previously analyzed, where it is can be seen that this design based on transmission holographic gratings is capable of accomplishing its task.

## 5. Conclusions

In this article, an optical see-through display based on a holographic waveguide has been designed and manufactured. To optimize the use of photopolymers as recording materials for this particular application, the device has been designed using slanted transmission diffraction gratings. Using a polyvinyl alcohol acrylamide (PVA/AA), in which BMA has been included in its composition, it has been shown that the index modulations necessary to obtain high diffraction efficiency can be obtained in the case of non-slanted diffraction gratings of 1000 and 2000 lines/mm, as well as in slanted diffraction gratings of 1700 lines/mm. Finally, the device designed using 1700 lines/mm slanted diffraction gratings has been manufactured, demonstrating its functionality.

## Figures and Tables

**Table 1:** Composition of the liquid solution for photopolymer AA.

TEA (mL)	PVA (mL) (8% *w*/*v*)	AA (g)	BMA (g)	YE (0.8% *w*/*v*) (mL)
2.0	25	0.84	0.25	0.7
